# Potential Biological and Genetic Links Between Dementia and Osteoporosis: A Scoping Review

**DOI:** 10.3390/geriatrics10040096

**Published:** 2025-07-20

**Authors:** Abayomi N. Ogunwale, Paul E. Schulz, Jude K. des Bordes, Florent Elefteriou, Nahid J. Rianon

**Affiliations:** 1 Department of Family and Community Medicine, UTHealth McGovern Medical School, Houston, TX 77030, USA; abayomi.n.ogunwale@uth.tmc.edu; 2 Department of Neurology, UTHealth McGovern Medical School, Houston, TX 77030, USA; paul.e.schulz@uth.tmc.edu; 3 Joan and Stanford Alexander Division of Geriatric and Palliative Medicine, Department of Internal Medicine, UTHealth McGovern Medical School, Houston, TX 77030, USA; jude.k.desbordes@uth.tmc.edu; 4 Department of Molecular and Human Genetics, Baylor College of Medicine, Houston, TX 77030, USA; florent.elefteriou@bcm.edu

**Keywords:** dementia, osteoporosis, biomarkers, genetic markers, scoping review

## Abstract

Background: The biological mediators for the epidemiologic overlap between osteoporosis and dementia are unclear. We undertook a scoping review of clinical studies to identify genetic and biological factors linked with these degenerative conditions, exploring the mechanisms and pathways connecting both conditions. Methods: Studies selected (1) involved clinical research investigating genetic factors or biomarkers associated with dementia or osteoporosis, and (2) were published in English in a peer-reviewed journal between July 1993 and March 2025. We searched Medline Ovid, Embase, PsycINFO, the Cochrane Library, the Web of Science databases, Google Scholar, and the reference lists of studies following the guidelines for Preferred Reporting Items for Systematic Reviews and Meta-Analyses for Scoping Reviews (PRISMA-ScR). Results: Twenty-three studies were included in this review. These explored the role of the APOE polymorphism (n = 2) and the APOE4 allele (n = 13), associations between TREM2 mutation and late onset AD (n = 1), and associations between amyloid beta and bone remodeling (n = 1); bone-related biomarkers like DKK1, OPG, and TRAIL as predictors of cognitive change (n = 2); extracellular vesicles as bone–brain communication pathways (1); and the role of dementia-related genes (n = 1), AD-related CSF biomarkers (n = 1), and parathyroid hormone (PTH) (n = 1) in osteoporosis–dementia pathophysiology. Conclusions: Bone-related biomarkers active in the Wnt/β-Catenin pathway (Dkk1 and sclerostin) and the RANKL/RANK/OPG pathway (OPG/TRAIL ratio) present consistent evidence of involvement in AD and osteoporosis development. Reports proposing APOE4 as a causal genetic link for both osteoporosis and AD in women are not corroborated by newer observational studies. The role of Aβ toxicity in osteoporosis development is unverified in a large clinical study.

## 1. Introduction

Osteoporosis and dementia are multifactorial degenerative conditions in older adults which exert considerable costs on healthcare systems worldwide. The cost of managing dementia is projected to rise to USD 1.5 trillion in the United States (US) by 2050 [1]. Similarly, the direct and indirect costs of osteoporosis in the US are estimated to reach USD 95 billion by 2040 [2]. An association between osteoporosis and dementia has long been suggested by basic and clinical research [3,4]; however, the pathways underpinning this relationship are unclear. Due to the overlap in the risk factors and demography of individuals affected by these conditions, multiple hypotheses have been proposed, and many potential biological risk factors have been investigated, including estrogen exposure [5,6], vitamins D [7] and K [8], the APOE4 allele [9], and amyloid beta (Aβ) [10]. A scoping review approach is selected for this review due to the novelty and breadth of the subject matter.

The objectives of this review are as follows:

i. To explore current clinical evidence and identify genetic and biological factors that may clarify the relationship between the degenerative processes in dementia and osteoporosis.
ii. To explore the potential mechanisms and biological pathways by which these genetic and biological factors may mediate the relationship between dementia and osteoporosis.

## 2. Materials and Methods

### 2.1. Eligibility

The inclusion criteria for studies in this review were as follows: (i.) clinical research investigating any genetic or biological factor relating to the pathologic relationship between dementia and osteoporosis, (ii.) publication in a peer-reviewed journal between July 1993 and March 2025, (iii.) written in English, and (iv.) available in full text. As we focused on the clinical relevance of the association between dementia and osteoporosis, we did not include preclinical studies in this review. In addition, we examined biological links and not just simple associations of these diseases with behavioral and lifestyle factors to streamline this exploratory project and highlight mediators with clinical utility.

### 2.2. Data Sources and Search Strategy

This scoping review was conducted using the Preferred Reporting Items for Systematic Reviews and Meta-Analyses for Scoping Reviews (PRISMA-ScR) guidelines. The protocol was registered in the Open Science Framework (OSF) (doi; 10.17605/OSF.IO/DB84X). We searched Medline (Ovid) as the primary database. First, we performed an initial search on 4 February 2022 and updated searches on 2 February 2024 and 20 March 2025. Supplementary Table S1 provides the detailed strategy for the search in Medline (Ovid). The search was then translated and conducted in the Embase, PsycInfo, Web of Science, and Cochrane databases. We updated the Cochrane search to match the syntax changes in that database. All other searches were identical between the initial search and the update. We searched using the MeSH terms ‘Aged (65 and over)’, ‘Osteoporosis’, and ‘Dementia’, all combined with related keyword terms: ‘aged’, ‘elderly’, ‘retiree’, ‘senior citizen’, ‘nursing home resident’, ‘osteoporosis’, ‘bone loss’, ‘dementia’, ‘Alzheimer’, and ‘other diseases with dementia’. Next, we searched the list of references of relevant articles and Google Scholar for additional qualifying studies. Duplicates of studies were removed.

### 2.3. Study Selection

Two authors (A.N.O and N.J.R) independently screened the abstracts. Full texts of the selected articles were retrieved and reviewed. A third author (J.K.dB) acted as a tie breaker in cases of disagreement. Reports meeting the inclusion criteria were included in the scoping review.

### 2.4. Data Charting

Data were extracted by one of the authors (AO) and reviewed by another (NR). Data extracted included information on the author, year of publication, biological or genetic mediators, study questions, and conclusions.

### 2.5. Data Analysis or Summary

Selected studies were summarized with emphasis on the genetic and biological markers which have an association with dementia or osteoporosis. In addition, we described evidence of how these biological and genetic factors potentially link dementia and osteoporosis through their effects on either bone, neural tissue, or both. We then created two tables summarizing the factors potentially connecting dementia and osteoporosis with a biological/pathophysiological rationale.

## 3. Results

**Figure 1:**
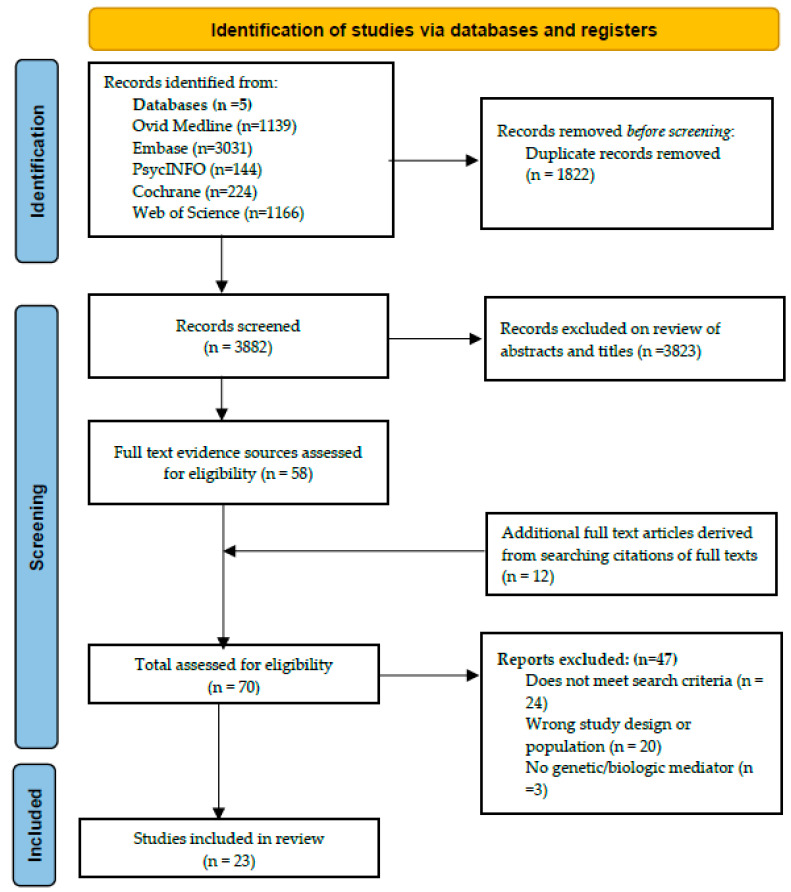
PRISMA flow diagram for study selection.

The search of databases yielded a total of 3882 de-duplicated results. A manual search of Google Scholar and references of studies yielded an additional 24 abstracts. In total, 3906 abstracts were reviewed and 3836 of these were excluded. Seventy full text articles were reviewed and of these, 23 clinical studies were included in the final review [10,11,12,13,14,15,16,17,18,19,20,21,22,23,24,25,26,27,28,29,30,31,32]. Figure 1 is a flowchart describing the selection process for the studies included in this scoping review.

Supplementary Table S2 is a summary of the studies included in this review.

Table 1 summarizes the factors with possible links to the degenerative processes occurring in the brain and bone in older adults.

## 4. Discussion

We discuss the 23 clinical studies reviewed as follows: (i.) evidence linking established dementia-related biomarkers with bone degeneration and (ii.) evidence linking bone-related biomarkers with cognitive changes.

### 4.1. Biomarkers with Known Link with or Predictive of Dementia

#### 4.1.1. Apolipoprotein E (APOE)

Fifteen of the twenty-three clinical studies included in this review examined the role of the APOE gene on bone homeostasis [12,13,14,18,19,30,31]. The APOE protein has vital roles in lipid metabolism, transport, and neuronal homeostasis [33,34,35]. APOE exists as three genetic isoforms (APOE2, E3, and E4) that are direct products of the three alleles of the APOE gene—ε2, ε3, and ε4. These alleles may combine into six genotypes (ε2/2, ε3/3, ε3/2, ε4/4, ε4/3, and ε4/2) depending on the gene copy inherited from each parent. APOE4 is present in about 20% of the general population and 65% of individuals with late onset AD [36] and is recognized as a major risk for late-onset familial and sporadic AD via its effect on amyloid deposition [34,36]. Current evidence suggests that the APOE genetic variants impact cognitive changes and AD risks widely via both Aβ-dependent and independent pathways [37].

In 1997, Shiraki et al. reported a significant association between homozygous APOE ε4 status and predicted lumbar Z scores in 284 postmenopausal Japanese women [19]. Study participants were divided into three groups based on the number of ε4 alleles present: APOE4 -/- (individuals with genotypes ε3/ε3 and ε3/ε2), APOE4 +/- (ε4/ε3 and ε4/ε2), and APOE4 +/+ (ε4/ε4). They observed a gene-dose effect of the ε4 allele on lumbar and total body BMD and Z scores. Although measured serum osteocalcin was higher in the ε4/ε4 group, an inference could not be made regarding the effect of the ε4 allele on the rate of bone turnover because of the relatively low number of ε4 homozygous individuals in that cohort. In a larger (n = 1750) longitudinal study to evaluate the effect of the APOE ε4 allele on fracture risk, BMD, and the rate of bone loss, Cauley et al. also reported that risk for hip fracture in women with at least one APOE ε4 allele was twice as high as those without [12]. Likewise, wrist fracture risk was higher in women with the APOE ε4 allele compared to those without [12]. Hip BMDs were lower in women with at least one APOE ε4 allele but the results were not statistically significant [12]. There was a slight reduction in risk estimates after controlling for falls, cognition, and calcaneal BMD. Nevertheless, these observations supported the notion of the APOE4 allele being a causal genetic determinant for both bone loss and AD [12]. Of note however, in that study, women with the APOE4 allele recorded faster weight loss and were more likely to have mothers with reported fractures at ages > 50 years, confounding variables which independently increase fracture risk. Also, estimated fracture risk was significantly higher in women having three or more additional risk factors—other than AD—for hip fracture, raising concerns about the relative contribution of the APOE4 allele vs. other risk factors for hip fracture (including age > 70 years, health status, a maternal history of fractures, medications, exercise, a history of falls, limitations of activities of daily living, and BMD) to overall fracture risk [12].

Dick et al. also reported an association between the presence of at least one APOE4 allele and quantitative ultrasound (QUS) measures at the lower calcaneus and greater trochanter of the hip bone [13]. QUS measures and BMD were slightly higher at the total hip, trochanteric, and intertrochanteric regions, but not the femoral neck in participants with no APOE4 allele. Individuals with the APOE4 allele were also more likely to have BMD in the osteopenia range. However, there was no association between APOE4 and biochemical indicators of bone remodeling (serum osteocalcin, calcium, phosphorus and alkaline phosphatase, and urine deoxypyridinoline/creatinine ratio) as well as fracture prevalence, raising questions about whether and how the APOE4 allele mediates its inhibitory effect on bone.

Johnston et al. reported a doubled risk of fractures in women with APOE4 compared to those without, after controlling for falls and dementia in a nested case–control study (n = 899) within the Monongahela Valley Independent Elders Survey (MoVIES) study [16]. The study design and reliance on self-reports for hip fracture cases in this study introduce bias and limit its validity. Similarly, Kohlmeier et al. reported a non-statistically significant increased risk of bone fracture in hemodialysis patients with at least one APOE4 allele compared to those without [17]. Although more than half of the fracture incidents occurred before the initiation of dialysis, the progressive impact of chronic renal impairment on bone metabolism and the small sample size (n = 219) limit the validity and generalizability of these results. Pluijm et al. reported lower femoral neck and trochanter BMD and higher vertebral deformities among women participants, independent of age [9]. Among male participants, an association between APOE4 status and lower hip BMD was only observed among younger men aged 65–69 years [9]. Respondents to this survey were, however, more likely to be younger and less frail, so able to visit the hospital for qualitative ultrasound. Souza et al. reported an association between the presence of APOE2 and APOE4 and lower BMD and higher serum C terminus collagen peptide and urinary deoxypyridinoline [20]. The small cohort sample (n = 413) limits the generalizability of these results.

Six studies in this review do not support the associations between APOE4 and bone loss/fragility. von Mühlen et al. reported no differences in the BMD at the hip, lumbar spine, or distal and midshaft radius of cohort members with and without the APOE ε4 allele, at baseline and following 4 years of follow-up in the Rancho Bernardo study (n = 928, ages 45–95 years) [30]. Efstathiadou also reported no association between APOE polymorphisms and BMD in a cohort of peri- and post-menopausal Greek women (n = 147, ages 35–76 years) [14]. Similarly, Wong et al. showed no association between the APOE4 gene and Z scores of femoral neck BMD in a cohort of Chinese and Japanese women (n = 457, ages 55–79 years) [31]. Booth et al. found no association between the E4 allele and both BMD and femoral fracture risk in 335 men (mean age 75.1 ± 4.93 years) and 553 women (mean age 75.3 ± 4.83 years) enrolled in the Framingham Heart Study [11]. Similarly, Heikkinen et al. found no association between the presence of the E4 allele and lumbar and femoral neck BMD as well as baseline concentrations of bone biomarkers (osteocalcin, type I collagen carboxy-terminal telopeptide (ICTP), and bone-specific alkaline phosphatase (BAP) in 352 postmenopausal women (ages 47–56 years) recruited from the Kuopio Osteoporosis Risk Factor and Prevention Study [15], and Schoofs et al. found no association between APOE genotypes and BMD, bone loss, and the rate of wrist and hip fractures in the Rotterdam study (n = 5857, mean age 69.3 years) [18]. Studies reporting a positive association between the APOE4 allele and a lower BMD and increased fracture risk have been in older women—average ages of 64 years [31], 71 years [12], 75 years [13], 76 years [16], and 75 years [9]—suggesting that sex and age may play a role in this relationship. Other criticisms against the validity of these studies include sample sizes of <1000 participants [12,16,17,19] and population genotype distribution not in Hardy–Weinberg equilibrium [9,12,17].

Lastly, a meta-analysis using data from seventeen studies, including individual-level data from the Framingham Heart Study (n = 1495) and the Vitamin K trial (n = 377), reported no association between the APOE4 allele and hip and total body BMD [38]. The initially observed statistical significance between the APOE4 allele and trochanteric and lumbar spine BMD disappeared following meta-regression and sensitivity analyses [38]. Therefore, prevailing evidence suggests that earlier reported associations between the APOE4 allele and bone outcomes, including BMD and fracture risk, are not consistently reproducible, and may reflect population-specific characteristics which have not been identified. Table 2 is a summary of the studies investigating the association between APOE and bone changes.

#### 4.1.2. Endogenous Aβ and Amyloid Precursor Protein (APP)

APP and its precursors, including Aβ precursor-like proteins 1 and 2 (APLP1 and APLP2), belong to a multigene family of transmembrane glycoproteins which have been implicated in AD [39]. In the amyloid cascade hypothesis of AD development, the accumulation of Aβ, a 40–42 amino acid peptide produced in the brain by the breakdown of APP by β and γ secretase, is a central step in AD pathogenesis [40]. Aβ exists in the body in two major alloforms: Aβ40 and Aβ42. Aβ40 is more abundant, but amyloid plaques seen in AD contain more Aβ42 [41,42]. Evidence linking these peptides to osteoclastogenesis has led to investigations of their potential roles in osteoporosis development. Stapledon et al. reported a correlation between levels of AD-related genes–including APP, APLP2, β-site amyloid precursor protein cleaving enzyme 1 (BACE1), and nerve growth factor (NGF)—and genes involved in the bone remodeling process—RANK and tartrate-resistant acid phosphatase (TRAP)—as well as the RANK/OPG mRNA ratio in patients admitted with a fracture of the neck of the femur [28]. These observations suggested a functional relationship between genes driving bone and brain degeneration around the period of the fracture, but no evidence for a causal relationship between both processes.

Physiologic levels of Aβ have been linked to the maintenance of synaptic activity, excitability, and cell survival. The concept of Aβ ‘toxicity’ arises when Aβ synthesis and clearance become unregulated, leading to the formation of plaques, as seen in AD. Although most research related to Aβ focuses on the brain, Li et al. examined bone tissues from 37 patients [29] with osteoporosis and 16 with osteopenia and 8 controls for evidence of pathologic Aβ deposits [43]. They reported significant elevations in levels of mRNA and expressed Aβ42 and APP protein in bone tissues from patients compared to age- and sex-matched healthy controls [43]. Furthermore, the expression levels for Aβ42 and APP were negatively correlated to BMD. The small size of this study’s total population (n = 45) as well as the small number of normal controls (n = 8), however, limit the accuracy and generalizability of these results. Pan et al. reported a positive association between osteoporosis and cortical and hippocampal atrophy over time, suggesting a link between decreased brain volume (as seen in dementia) and low bone mass. Initial associations between osteoporosis and baseline titers of AD-related CSF biomarkers—CSF Aβ load, t-tau (total tau), or p-tau181 (tau with threonine-181 phosphorylation)—on cross-sectional analysis were, however, not sustained on longitudinal analysis [26]. The cross-sectional design of this study is a drawback. The analysis also excluded important confounders such as hypertension and hyperlipidemia, which may alter the interpretation of results.

The ratio of Aβ42 to Aβ40 (Aβ42/Aβ40) in plasma and CSF is recognized as a good index of cortical amyloid load. There is an inverse association between plasma Aβ42/Aβ40 and brain amyloid burden, and the progression and severity of cognitive decline [44]. Zhang et al. examined the relationship between markers of bone health (hip bone BMD) and cognition (Mini-Mental State Examination [MMSE] and Auditory Verbal Learning Test—delayed recall [AVLT-DR]) in older adults with and without osteopenia and AD. Individuals with osteopenia presented with significantly lower MMSE and AVL-DR scores and both plasma Aβ42 and Aβ42/Aβ40, compared to individuals without osteopenia. Following regression analysis, lower plasma Aβ42/Aβ40 and BMD were associated with lower MMSE and AVL-DR scores. On mediation analysis, among individuals with osteopenia, plasma Aβ42/Aβ40 served as a mediator in the interaction between BMD and both MMSE and AVL-DR scores. Similarly, individuals with AD recorded significantly lower BMD, MMSE, AVL-DR scores, CSF Aβ42, and CSF Aβ42/Aβ40 when compared to those without AD. On regression analysis, BMD correlated positively with both MMSE score and CSF Aβ42/Aβ40. Overall, these results highlight the potential of the hip bone BMD as an index of cognition, and furthermore, changes in plasma and CSF Aβ42/Aβ40 as a mediator between BMD and cognition [45]. In line with these results, it is postulated that excess Aβ exerts its effect on bone in a bone-autonomous manner via osteoclast activation [46].

#### 4.1.3. DKK1

The Wnt pathway is an evolutionarily conserved signaling pathway significant for the development, regeneration, and maintenance of homeostasis in the skeletal, vascular, and nervous systems [47]. Three pathways have been described: the canonical Wnt/β catenin, and noncanonical Wnt/Ca^2+^ and planar cell polarity pathways. Emerging evidence suggests the canonical pathway plays key roles in both bone and neuronal degeneration. Here, Wnt binds to the Frizzled (FZD) and lipoprotein receptor-related proteins 5 and 6 (LRP5/6) coreceptor to activate a signaling cascade which relocates cytoplasmic-bound β catenin glycoproteins from the cytosol to the nucleus for target gene transcription [47]. Dickopf-related protein 1 (DKK1), a glycoprotein secreted by osteoblasts and osteocytes, is an endogenous inhibitor of Wnt-LRP5/6 interaction and its downstream effects. DKK1 hypersecretion has been linked to focal osteolytic bone lesions in patients with multiple myeloma [48]. Furthermore, loss-of-function [49] and gain-of-function [50] mutations in the coreceptor gene, LRP5, have been linked to disorders characterized by low bone mass [49] and excessive bone mass [50], respectively.

Disruptions of the Wnt pathway have been implicated in Parkinson’s disease (PD) [48], multiple sclerosis [51], stroke [52], and AD pathogenesis [53]. Elevated levels of DKK1 have been reported in brain sections of late-stage AD patients, and the inhibition of the Wnt pathway by DKK1 has been linked to synaptic dysfunction [54], neuron apoptosis [55], and other Aβ-linked changes in mouse brains [54]. It has also been linked to blood–brain barrier integrity, which is of relevance to AD progression. Ross et al. examined the association between bone-related biomarkers and AD in community-dwelling individuals with memory concerns but no AD diagnosis [27]. They reported a correlation between baseline serum OPG, sclerostin, OPG/TRAIL ratio, OPG/RANKL ratio, and cognition which disappeared after adjustments for age, sex, and education. The association between DKK1 and the annual rate of change in cognition disappeared when patients who developed AD during the study period were excluded [27]. This is significant, as it appears to link DKK1 and cognition only via AD development. Reported positive associations between baseline TRAIL and the rate of cognition change (*p* < 0.001) as well as a negative association between the OPG/TRAIL ratio and rate of cognition change (*p* < 0.003) were sustained after adjusting for the number of visits [27]. The enrolment of individuals with preexisting memory concerns raises potential for selection bias. Cohort size was small and mostly female, and the narrow range of demographic variables including age and global cognition scores, effect of retesting on cognition scores, and timing of collection of the biomarkers are limitations that affect the accuracy and generalizability of these results. Another study by Tay et al. in individuals with mild cognitive impairment and mild to moderate AD also showed a rise in serum DKK1 in participants with objective AD progression compared to lower serum DKK1 in non-progressors, suggesting a role for DKK1 in progression from mild cognitive impairment to AD [56]. Again, these results have limitations related to the risk of selection bias and unknown confounders that were unaccounted for. These human studies highlight a mechanism by which Aβ toxicity on neurons is potentially mediated via DKK1’s modulation of the Wnt pathway in the CNS, but they also raise questions about the significance of Aβ presence in the cascade of events, and the origin and role of DKK1 if any, in the subset of dementia patients without brain Aβ accumulation.

Sclerostin, an osteocyte-specific glycoprotein that inhibits the canonical Wnt pathway (similarly to DKK1 by binding to LRP5/6 [57]) is the target of romosozumab, the humanized monoclonal antibody treatment for osteoporosis [58]. A recent study demonstrates elevations in plasma sclerostin in cognitively unimpaired individuals, with higher levels seen with those having higher brain Aβ on PET scans. Serum sclerostin was shown to be correlated to the Aβ load, suggesting a potential role for sclerostin as a biomarker for AD [59]. A recent study reports a lower risk of Parkinson’s disease in older adults with osteoporosis who were treated with romosozumab compared to those treated with two parathyroid hormone receptor agonists—teriparatide and abaloparatide—suggesting that Wnt/catenin pathway activation via the blocking of sclerostin may reduce the risk of PD progression from the prodromal to the motor stage [60]. The low number of participants who develop PD is a limitation. Among the PD events, there was also no testing to rule out the misdiagnosis of other types of PD, including drug-induced PD and vascular PD [60]. Currently, there is insufficient clinical data to confirm that sclerostin crosses the blood–brain barrier and inhibits the Wnt-catenin pathway in the human brain (although it does experimentally in preclinical models) [61].

### 4.2. Biomarkers with Bone and Brain Tissue-Autonomous Effects

#### 4.2.1. OPG and TRAIL

OPG or tumor necrosis factor (TNF) receptor superfamily member 11B, or osteoclast inhibitory factor (OIF), is a soluble glycoprotein receptor secreted by osteoblasts, osteogenic stromal stem cells, B cells, and dendritic cells [62]. It affects osteoclastogenesis by acting as a decoy receptor for the interaction between the receptor activator of nuclear factor kappa B ligand (RANKL) and receptor activator of nuclear factor kappa B (RANK) [63]. Low OPG expression, elevated OPG expression (in the presence of low RANKL), and a low OPG/RANKL ratio have been associated with post-menopausal osteoporosis [64] and fragility fractures; elevated OPG has also been associated with atherosclerosis [65], coronary artery disease [66], heart failure [67], microvascular changes in diabetes mellitus and chronic kidney disease [68,69], and primary biliary cirrhosis [70].

To build on the reported associations between atherosclerosis, cardiovascular disease and dementia, Emanuele et al. investigated OPG’s potential as a biomarker for vascular dementia (VaD) and AD in a case–control study [22]. They recorded higher serum OPG in participants with VaD than in those with AD, with both populations having higher OPG levels than controls, postulating that these differences may reflect the level of atherosclerosis in individuals across these groups. Logistics regression analysis showed independent associations between OPG levels and both VaD and AD risks after controlling for age, gender, and the presence of the APOE4 allele [22]. The validity of these results is, however, limited by the case–control design of this study, and multiple unmeasured and uncontrolled confounders in the analysis. As OPG is expressed by multiple organ systems, it is arguable that the elevated serum levels noted in this study are not solely due to VaD or AD disease states. Also, in a population at high risk for osteoporosis—which is also associated with elevated OPG—such as this one, DEXA measurements of BMD were not taken, thereby leaving a potential confounder unaddressed. Luckhaus et al. reported no difference in OPG level between individuals with AD and MCI compared to controls [24], but the small size of that study population is a limitation. Currently, our understanding of the exact role of OPG in AD pathogenesis remains unclear.

An understanding of the role of the OPG–RANK–RANKL axis in bone homeostasis formed the fulcrum for the development of Denosumab, the human monoclonal antibody against RANKL with antiresorptive activity. While the RANK–RANKL interaction has been shown to be catabolic in the skeletal system (via osteoclasts), it appears to have an inhibitory effect on neuroinflammation, mainly via toll-like receptors 3 (TLR3). Alone, an OPG titer is an unreliable predictor of bone loss, with both low [71] and elevated OPG [72] expressions previously linked to low BMD and vertebral fragility fractures. The OPG/RANKL ratio appears to be a better indicator of the direction of bone remodeling [73]. There is evidence that under ischemic stress, OPG, secreted by oligodendrocytes and neurons, may be neurotoxic both directly by its proinflammatory effect and indirectly by its inhibition of RANK/RANKL coupling in microglial cells [74]. The significance of the OPG/RANKL ratio in dementia prediction and pathogenesis has not been similarly demonstrated.

Tumor necrosis factor (TNF)-related apoptosis-inducing ligand (TRAIL), also known as TNF Ligand Superfamily Member 10 or cluster of differentiation 253 (CD253), is a cytokine with pro-apoptotic activities when bound to death receptor 4 (DR4 or TRAILR1) and death receptor 5 (DR5 or TRAILR2). TRAIL is pro-osteoclastogenic by blocking the inhibitory effect of OPG on RANK–RANKL interaction [75,76]. Ross et al. reported associations between serum titers of TRAIL (*p* < 0.001) and other biomarkers, including DKK1 (*p* = 0.014) and CTX-1 (*p* = 0.046), and the annual rate of change in global cognition [27]. However, beyond its role in osteoclast activation and as a potential index for the rate of cognitive change, the role of TRAIL in dementia and osteoporosis pathogenesis remains unclear.

#### 4.2.2. Osteocyte-Derived and Brain-Derived Extracellular Vesicles

Liu et al. demonstrated the transportation of brain-derived EVs administered both via intracerebroventricular and intravenous routes to bone tissue in both AD patients and mice [23]. Both brain-derived and plasma-derived EVs from AD patients were associated with impaired osteogenesis and increased bone fat deposition in vitro. This study highlights the existence of additional, novel pathways for bone and brain communication, and how dementia may impact osteoporosis. They say little about the biological events preceding this cellular and biochemical cascade of interactions.

#### 4.2.3. Parathyroid Hormone

The serum parathyroid hormone (PTH) level rises with advancing age and has a vital role in the homeostasis of both serum vitamin D and calcium [77,78]. Braverman et al. examined the association between serum PTH levels and both bone density and a measure of neurologic processing speed—P300—in an age-matched sample of patients aged 18–90 years (n = 92, mean age 58.85 ± 15.47 years). They found statistically significant associations between serum PTH titer > 30 pg/mL and slower P300 and lower bone density, suggesting a role for PTH in the pathologic process for both conditions [21]. In this analysis, however, multiple factors affecting serum PTH including age, sex, menopausal status, the use of vitamin D, and calcium supplements were not controlled. Kim et al. examined the association between dementia and serum PTH levels using data from the prospective Atherosclerosis Risk in Communities (ARIC) Health Cohort and found no statistically significant changes in global cognitive scores [79].

#### 4.2.4. Triggering Receptor Expressed on Myeloid Cells 2 (TREM2) R47H

Guerreiro et al. employed multiple epidemiologic strategies to investigate the association between the TREM 2 R47H mutation and AD, including the genome sequencing of 1092 AD cases and 1107 controls, a meta-analysis of three genome-wide studies (the AddNeuroMed (ANM), Genetic and Environmental Risk for Alzheimer’s Disease Consortium (GERAD), and the European Alzheimer’s Disease Initiative Consortium (EADI) studies), and direct genotyping in 1887 AD cases with R47H genotypes and 4061 controls [80]. The study identified six variants of TREM2 present in the AD cases compared to controls (T66M, H157Y, D87N, Q33X, R98W, and Y38C) [80]. The meta-analysis step showed that the R47H variant had the strongest association with AD. The R47H variant was selectively genotyped in the participants of the case–control series. Logistic regression analysis showed a significant association between the presence of the R47h variant and AD [80]. This approach, however, raises concerns about reproducibility, unaddressed biases, and confounders from the studies in the meta-analysis. Jonsson et al. employed a similar stepwise approach including the genome sequencing of 2261 Icelanders to identify suspect sequence variants and the comparison of these variants in AD patients and controls to investigate associations between these variants and AD, and thereafter replicated the same investigation in a series of case–control studies on individuals from Norway, the Netherlands, Germany, and the US [81]. The case–control design is a limitation. In combining the results from these different populations to determine an overall association between the rs75932628-T TREM 2 variant and AD, errors from the individual studies were unaddressed. While it is biologically plausible that mutations in the TREM2 gene may impair microglial function and amyloid clearance in the brain, the rarity of these sequence variants encumbers the generalizability of these results.

Ma et al. caried out a study to evaluate the effect of the TREM2 polymorphism on late onset dementia in 279 AD patients and 346 controls drawn from a Chinese Southern Han population. None of the AD patients tested positive for the assayed TREM2 genotype (rs75932628), weakening support for a role for the TREM2 mutation in AD pathogenesis and as a link between osteoporosis and dementia [25]. So far, associations between TREM2 mutation and AD have only been verified in individuals of European and North American ancestry, with findings not being reproducible in cohorts of individuals of African, Chinese, and Japanese descent [25,82,83,84].

#### 4.2.5. ARHGEF15 Mutations

Rho guanine nucleotide exchange factor 15, also known as Ephexin 5 (E5), is a guanine nucleotide exchange factor specific for modulating the interaction of the ras homolog family member A (RhoA) gene with its effector molecules. ARHGEF15 is important for smooth muscle contractions and has been shown to be elevated in mouse hippocampal neurons exposed to Aβ1-42 [85]. Ding et al. presented evidence suggesting mutations in ARHGEF15 as a causal risk factor for cerebral small vessel disease (CSVD), a leading cause of vascular dementia (VaD) [86]. The study enrolled 232 individuals with CSVD and 15 controls. Patients with the ARHGEF15 mutations presented with lower BMD, higher fracture risk, and both postmortem and radiologic evidence of CSVD. In vitro experiments demonstrated disruptions of F actin cytoskeleton and osteoblast dysfunction upon the deletion of the gene [86]. However, VaD accounts for 15–20% of dementia cases only, and ARHGEF15 mutations do not explain the relationship between more prevalent types of dementia and osteoporosis.

### 4.3. Limitations

Our restriction of selected studies to only published studies in English from July 1993 to March 2025 also means that many published and all unpublished studies, and studies in other languages, have been excluded. The selection bias introduced may reduce the quality and the scope of our analyses. One other major limitation of this exploratory review is the exclusion of animal studies, which could strengthen the evidence for the biological mediators examined. These basic science studies could also have provided some context and helped clarify some of the findings in this clinical studies review. However, the biochemical elements at play in the overlap between the pathophysiology of dementia and osteoporosis have not been fully elucidated. The exclusion of the basic science studies therefore allows for a more streamlined review, with a focus on those mediators with clinical evidence. Another limitation is that the selected studies come from different populations and were conducted with varying methodologies, hence their different biases and errors translate to the scoping review. To reduce the effect of this, we have highlighted the biases in each study and the attempts to control them. Also, there is a relative dearth of published evidence to foster a more detailed examination of the role of PTH and ARHGEF15 mutation in the association between osteoporosis and dementia pathophysiology.

## 5. Conclusions

DKK1 and sclerostin are bone-related factors showing promise as potential biomarkers for dementia prediction. Current evidence implicates DKK1 in the amyloid-induced toxicity that underpins AD progression and highlights the emergence of sclerostin as a bone-related biomarker with potential for early dementia detection. Our review also found consistent evidence suggesting that the normal functioning of the Wnt-catenin pathway counters osteoporosis development (by stimulating osteoblast differentiation) and dementia development (by supporting synaptic activity, blood-brain integrity, and inhibiting Aβ formation and tau hyperphosphorylation). DKK1 and sclerostin also directly impact neurodegeneration and bone loss via their effects on the Wnt-catenin pathway. Future research may help clarify the temporal relationship between DKKI elevation and amyloid deposition. Research into the effect of anti-sclerostin antibodies such as romosozumab on AD development and dementia progression in individuals with MCI will be helpful.

Of the established dementia-related biomarkers examined in this review, only Aβ and APOE4 appear to be associated with bone remodeling. However, associations between APOE4 and both lower BMD and increased fracture risk are not consistently reported, suggesting that other factors may mediate this relationship, or they may be population specific.

This scoping review is hopefully a start to a deeper exploration of the mediators of the pathophysiologic overlap between bone and brain degeneration in older adults. Future targeted reviews and preclinical studies will be critical to address in more detail the mechanistic aspects of these associations between dementia/AD and osteoporosis.

## Figures and Tables

**Table 1:** Biomarker or gene with evidence of dementia and osteoporosis involvement.

Biomarker of Gene	Association with Dementia	Association with Osteoporosis
APOE4	+	inconsistent
OPG	-	+
OPG/RANKL ratio	-	+
TRAIL	inconsistent	inconsistent
TREM2 mutations	inconsistent	-
PTH	-	+
ARHGEF15	inconsistent	-
DKK1	+	+
Sclerostin	+	+
Amyloid β	+	+
OPG/Trail ratio	+	+

+: established association. -: No established association.

**Table 2:** Summary of studies examining the association between APOE status and bone changes.

Study	Total Study Participants	Association with BMD/Osteoporosis/Fracture
Booth [11]	888	No
Cauley [12]	1750	Yes
Dick [13]	1332	Yes
Efstathiadou [14]	147	No
Heikkinen [15]	352	No
Johnston [16]	899	Yes
Kohlmeier [17]	219	Yes
Pluijm [9]	604	No
Schoofs [18]	5857	No
Shiraki [19]	284	Yes
Souza [20]	529	Yes
Wong [22]	692	No
von Mühlen [23]	596	No

## Data Availability

The raw data supporting the conclusions of this article will be made available by the authors on request.

## Supplementary Materials

The following supporting information can be downloaded at https://www.mdpi.com/article/10.3390/geriatrics10040096/s1: Table S1. Search Strategy Employed in Primary Database (Ovid Medline) and Table S2. Summary of Clinical Studies Included in the Review.

## References

[1] Zissimopoulos J., Crimmins E., St Clair P. (2014). The Value of Delaying Alzheimer’s Disease Onset. Forum Health Econ. Policy.

[2] Lewiecki E.M., Ortendahl J.D., Vanderpuye-Orgle J., Grauer A., Arellano J., Lemay J., Harmon A.L., Broder M.S., Singer A.J. (2019). Healthcare Policy Changes in Osteoporosis Can Improve Outcomes and Reduce Costs in the United States. JBMR Plus.

[3] Baker N.L., Cook M.N., Arrighi H.M., Bullock R. (2011). Hip fracture risk and subsequent mortality among Alzheimer’s disease patients in the United Kingdom, 1988–2007. Age Ageing.

[4] Friedman S.M., Menzies I.B., Bukata S.V., Mendelson D.A., Kates S.L. (2010). Dementia and hip fractures: Development of a pathogenic framework for understanding and studying risk. Geriatr. Orthop. Surg. Rehabil..

[5] Bove R., Secor E., Chibnik L.B., Barnes L.L., Schneider J.A., Bennett D.A., De Jager P.L. (2014). Age at surgical menopause influences cognitive decline and Alzheimer pathology in older women. Neurology.

[6] Tan Z.S., Seshadri S., Beiser A., Zhang Y., Felson D., Hannan M.T., Au R., Wolf P.A., Kiel D.P. (2005). Bone mineral density and the risk of Alzheimer disease. Arch. Neurol..

[7] Littlejohns T.J., Henley W.E., Lang I.A., Annweiler C., Beauchet O., Chaves P.H., Fried L., Kestenbaum B.R., Kuller L.H., Langa K.M. (2014). Vitamin D and the risk of dementia and Alzheimer disease. Neurology.

[8] Elkattawy H.A., Ghoneim F.M., Eladl M.A., Said E., Ebrahim H.A., El-Shafey M., Asseri S.M., El-Sherbiny M., Alsalamah R.H., Elsherbiny N.M. (2022). Vitamin K2 (Menaquinone-7) Reverses Age-Related Structural and Cognitive Deterioration in Naturally Aging Rats. Antioxidants.

[9] Pluijm S.M., Dik M.G., Jonker C., Deeg D.J., van Kamp G.J., Lips P. (2002). Effects of gender and age on the association of apolipoprotein E epsilon4 with bone mineral density, bone turnover and the risk of fractures in older people. Osteoporos. Int..

[10] Lin Y., Chen T., Chen J., Fang Y., Zeng C. (2021). Endogenous Aβ induces osteoporosis through an mTOR-dependent inhibition of autophagy in bone marrow mesenchymal stem cells (BMSCs). Ann. Transl. Med..

[11] Booth S.L., Tucker K.L., Chen H., Hannan M.T., Gagnon D.R., Cupples L.A., Wilson P.W., Ordovas J., Schaefer E.J., Dawson-Hughes B. (2000). Dietary vitamin K intakes are associated with hip fracture but not with bone mineral density in elderly men and women. Am. J. Clin. Nutr..

[12] Cauley J.A., Zmuda J.M., Yaffe K., Kuller L.H., Ferrell R.E., Wisniewski S.R., Cummings S.R. (1999). Apolipoprotein E polymorphism: A new genetic marker of hip fracture risk--The Study of Osteoporotic Fractures. J. Bone Miner. Res..

[13] Dick I.M., Devine A., Marangou A., Dhaliwal S.S., Laws S., Martins R.N., Prince R.L. (2002). Apolipoprotein E4 is associated with reduced calcaneal quantitative ultrasound measurements and bone mineral density in elderly women. Bone.

[14] Efstathiadou Z., Koukoulis G., Stakias N., Challa A., Tsatsoulis A. (2004). Apolipoprotein E polymorphism is not associated with spinal bone mineral density in peri- and postmenopausal Greek women. Maturitas.

[15] Heikkinen A.M., Kröger H., Niskanen L., Komulainen M.H., Ryynänen M., Parviainen M.T., Tuppurainen M.T., Honkanen R., Saarikoski S. (2000). Does apolipoprotein E genotype relate to BMD and bone markers in postmenopausal women?. Maturitas.

[16] Johnston J.M., Cauley J.A., Ganguli M. (1999). APOE 4 and hip fracture risk in a community-based study of older adults. J. Am. Geriatr. Soc..

[17] Kohlmeier M., Saupe J., Schaefer K., Asmus G. (1998). Bone fracture history and prospective bone fracture risk of hemodialysis patients are related to apolipoprotein E genotype. Calcif. Tissue Int..

[18] Schoofs M.W., van der Klift M., Hofman A., van Duijn C.M., Stricker B.H., Pols H.A., Uitterlinden A.G. (2004). ApoE gene polymorphisms, BMD, and fracture risk in elderly men and women: The Rotterdam study. J. Bone Miner. Res..

[19] Shiraki M., Shiraki Y., Aoki C., Hosoi T., Inoue S., Kaneki M., Ouchi Y. (1997). Association of bone mineral density with apolipoprotein E phenotype. J. Bone Miner. Res..

[20] Souza L.S., Rochette N.F., Pedrosa D.F., Magnago R.P.L., Filho T.B.F., Vieira F.L.H., Fin I.D.C.F., Eis S.R., Graceli J.B., Rangel L.B. (2018). Role of APOE Gene in Bone Mineral Density and Incidence of Bone Fractures in Brazilian Postmenopausal Women. J. Clin. Densitom..

[21] Braverman E.R., Chen T.J., Chen A.L., Arcuri V., Kerner M.M., Bajaj A., Carbajal J., Braverman D., Downs B.W., Blum K. (2009). Age-related increases in parathyroid hormone may be antecedent to both osteoporosis and dementia. BMC Endocr. Disord..

[22] Emanuele E., Peros E., Scioli G.A., D’Angelo A., Olivieri C., Montagna L., Geroldi D. (2004). Plasma osteoprotegerin as a biochemical marker for vascular dementia and Alzheimer’s disease. Int. J. Mol. Med..

[23] Liu X., Chen C., Jiang Y., Wan M., Jiao B., Liao X., Rao S., Hong C., Yang Q., Zhu Y. (2023). Brain-derived extracellular vesicles promote bone-fat imbalance in Alzheimer’s disease. Int. J. Biol. Sci..

[24] Luckhaus C., Mahabadi B., Grass-Kapanke B., Jänner M., Willenberg H., Jäger M., Supprian T., Fehsel K. (2009). Blood biomarkers of osteoporosis in mild cognitive impairment and Alzheimer’s disease. J. Neural Transm..

[25] Ma J., Zhou Y., Xu J., Liu X., Wang Y., Deng Y., Wang G., Xu W., Ren R., Liu X. (2014). Association study of TREM2 polymorphism rs75932628 with late-onset Alzheimer’s disease in Chinese Han population. Neurol. Res..

[26] Pan H., Cao J., Wu C., Huang F., Wu P., Lang J., Liu Y. (2022). Osteoporosis is associated with elevated baseline cerebrospinal fluid biomarkers and accelerated brain structural atrophy among older people. Front. Aging Neurosci..

[27] Ross R.D., Shah R.C., Leurgans S., Bottiglieri T., Wilson R.S., Sumner D.R. (2018). Circulating Dkk1 and TRAIL Are Associated With Cognitive Decline in Community-Dwelling, Older Adults With Cognitive Concerns. J. Gerontol. A Biol. Sci. Med. Sci..

[28] Stapledon C.J.M., Stamenkov R., Cappai R., Clark J.M., Bourke A., Bogdan Solomon L., Atkins G.J. (2021). Relationships between the Bone Expression of Alzheimer’s Disease-Related Genes, Bone Remodelling Genes and Cortical Bone Structure in Neck of Femur Fracture. Calcif. Tissue Int..

[29] Stefanidou M., O’Donnell A., Himali J.J., DeCarli C., Satizabal C., Beiser A.S., Seshadri S., Zaldy T. (2021). Bone Mineral Density Measurements and Association With Brain Structure and Cognitive Function: The Framingham Offspring Cohort. Alzheimer Dis. Assoc. Disord..

[30] von Mühlen D.G., Barrett-Connor E., Schneider D.L., Morin P.A., Parry P. (2001). Osteoporosis and apolipoprotein E genotype in older adults: The Rancho Bernardo study. Osteoporos. Int..

[31] Wong S.Y., Lau E.M., Li M., Chung T., Sham A., Woo J. (2005). The prevalence of Apo E4 genotype and its relationship to bone mineral density in Hong Kong Chinese. J. Bone Miner. Metab..

[32] Zhang P., Zhou Y., Chen G., Li J., Wang B., Lu X. (2022). Potential association of bone mineral density loss with cognitive impairment and central and peripheral amyloid-β changes: A cross-sectional study. BMC Musculoskelet. Disord..

[33] Hauser P.S., Narayanaswami V., Ryan R.O. (2011). Apolipoprotein E: From lipid transport to neurobiology. Prog. Lipid Res..

[34] Huang Y., Mahley R.W. (2014). Apolipoprotein E: Structure and function in lipid metabolism, neurobiology, and Alzheimer’s diseases. Neurobiol. Dis..

[35] Pang S., Li J., Zhang Y., Chen J. (2018). Meta-Analysis of the Relationship between the APOE Gene and the Onset of Parkinson’s Disease Dementia. Park. Dis..

[36] Saunders A.M., Strittmatter W.J., Schmechel D., George-Hyslop P.S., Pericak-Vance M., Joo S., Rosi B., Gusella J., Crapper-MacLachlan D., Alberts M. (1993). Association of apolipoprotein E allele epsilon 4 with late-onset familial and sporadic Alzheimer’s disease. Neurology.

[37] Raulin A.C., Doss S.V., Trottier Z.A., Ikezu T.C., Bu G., Liu C.C. (2022). ApoE in Alzheimer’s disease: Pathophysiology and therapeutic strategies. Mol. Neurodegener..

[38] Peter I., Crosier M.D., Yoshida M., Booth S.L., Cupples L.A., Dawson-Hughes B., Karasik D., Kiel D.P., Ordovas J.M., Trikalinos T.A. (2011). Associations of APOE gene polymorphisms with bone mineral density and fracture risk: A meta-analysis. Osteoporos Int..

[39] Johnston J.A., Norgren S., Ravid R., Wasco W., Winblad B., Lannfelt L., Cowburn R.F. (1996). Quantification of APP and APLP2 mRNA in APOE genotyped Alzheimer’s disease brains. Brain Res. Mol. Brain Res..

[40] Hardy J.A., Higgins G.A. (1992). Alzheimer’s disease: The amyloid cascade hypothesis. Science.

[41] Gu L., Guo Z. (2013). Alzheimer’s Aβ42 and Aβ40 peptides form interlaced amyloid fibrils. J. Neurochem..

[42] Iwatsubo T., Odaka A., Suzuki N., Mizusawa H., Nukina N., Ihara Y. (1994). Visualization of A beta 42(43) and A beta 40 in senile plaques with end-specific A beta monoclonals: Evidence that an initially deposited species is A beta 42(43). Neuron.

[43] Li S., Liu B., Zhang L., Rong L. (2014). Amyloid beta peptide is elevated in osteoporotic bone tissues and enhances osteoclast function. Bone.

[44] Fandos N., Pérez-Grijalba V., Pesini P., Olmos S., Bossa M., Villemagne V.L., Doecke J., Fowler C., Masters C.L., Sarasa M. (2017). Plasma amyloid β 42/40 ratios as biomarkers for amyloid β cerebral deposition in cognitively normal individuals. Alzheimers Dement..

[45] Zhang X.F., Attia J., D’Este C., Yu X.H., Wu X.G. (2005). A risk score predicted coronary heart disease and stroke in a Chinese cohort. J. Clin. Epidemiol..

[46] Cui S., Xiong F., Hong Y., Jung J.U., Li X.S., Liu J.Z., Yan R., Mei L., Feng X., Xiong W.C. (2011). APPswe/Aβ regulation of osteoclast activation and RAGE expression in an age-dependent manner. J. Bone Miner. Res..

[47] Komiya Y., Habas R. (2008). Wnt signal transduction pathways. Organogenesis.

[48] Liu Y., Hao S., Yang B., Fan Y., Qin X., Chen Y., Hu J. (2017). Wnt/β-catenin signaling plays an essential role in α7 nicotinic receptor-mediated neuroprotection of dopaminergic neurons in a mouse Parkinson’s disease model. Biochem. Pharmacol..

[49] Gong Y., Slee R.B., Fukai N., Rawadi G., Roman-Roman S., Reginato A.M., Wang H., Cundy T., Glorieux F.H., Lev D. (2001). LDL receptor-related protein 5 (LRP5) affects bone accrual and eye development. Cell.

[50] Little R.D., Carulli J.P., Del Mastro R.G., Dupuis J., Osborne M., Folz C., Manning S.P., Swain P.M., Zhao S.C., Eustace B. (2002). A mutation in the LDL receptor-related protein 5 gene results in the autosomal dominant high-bone-mass trait. Am. J. Hum. Genet..

[51] Vallée A., Vallée J.N., Guillevin R., Lecarpentier Y. (2018). Interactions Between the Canonical WNT/Beta-Catenin Pathway and PPAR Gamma on Neuroinflammation, Demyelination, and Remyelination in Multiple Sclerosis. Cell Mol. Neurobiol..

[52] Song S., Huang H., Guan X., Fiesler V., Bhuiyan M.I.H., Liu R., Jalali S., Hasan N., Tai A.K., Chattopadhyay A. (2021). Activation of endothelial Wnt/β-catenin signaling by protective astrocytes repairs BBB damage in ischemic stroke. Prog. Neurobiol..

[53] Vargas J.Y., Fuenzalida M., Inestrosa N.C. (2014). In vivo activation of Wnt signaling pathway enhances cognitive function of adult mice and reverses cognitive deficits in an Alzheimer’s disease model. J. Neurosci..

[54] Purro S.A., Dickins E.M., Salinas P.C. (2012). The secreted Wnt antagonist Dickkopf-1 is required for amyloid β-mediated synaptic loss. J. Neurosci..

[55] De Ferrari G.V., Chacón M.A., Barría M.I., Garrido J.L., Godoy J.A., Olivares G., Reyes A.E., Alvarez A., Bronfman M., Inestrosa N.C. (2003). Activation of Wnt signaling rescues neurodegeneration and behavioral impairments induced by beta-amyloid fibrils. Mol. Psychiatry.

[56] Tay L., Leung B., Yeo A., Chan M., Lim W.S. (2019). Elevations in Serum Dickkopf-1 and Disease Progression in Community-Dwelling Older Adults With Mild Cognitive Impairment and Mild-to-Moderate Alzheimer’s Disease. Front. Aging Neurosci..

[57] Li X., Zhang Y., Kang H., Liu W., Liu P., Zhang J., Harris S.E., Wu D. (2005). Sclerostin binds to LRP5/6 and antagonizes canonical Wnt signaling. J. Biol. Chem..

[58] Bandeira L., Lewiecki E.M., Bilezikian J.P. (2017). Romosozumab for the treatment of osteoporosis. Expert. Opin. Biol. Ther..

[59] Yuan J., Pedrini S., Thota R., Doecke J., Chatterjee P., Sohrabi H.R., Teunissen C.E., Verberk I.M.W., Stoops E., Vanderstichele H. (2023). Elevated plasma sclerostin is associated with high brain amyloid-β load in cognitively normal older adults. NPJ Aging.

[60] Inokuchi S., Shimamoto K. (2024). Wnt/β-catenin pathway as a potential target for Parkinson’s disease: A cohort study of romosozumab using routinely collected health data in Japan. Front. Pharmacol..

[61] Shi T., Shen S., Shi Y., Wang Q., Zhang G., Lin J., Chen J., Bai F., Zhang L., Wang Y. (2024). Osteocyte-derived sclerostin impairs cognitive function during ageing and Alzheimer’s disease progression. Nat. Metab..

[62] Simonet W.S., Lacey D.L., Dunstan C.R., Kelley M., Chang M.S., Luthy R., Nguyen H.Q., Wooden S., Bennett L., Boone T. (1997). Osteoprotegerin: A novel secreted protein involved in the regulation of bone density. Cell.

[63] Boyce B.F., Xing L. (2007). Biology of RANK, RANKL, and osteoprotegerin. Arthritis Res. Ther..

[64] Veshnavei H.A. (2022). Evaluation of the serum level of osteoprotegerin and bone mineral density in postmenopausal women. Int. J. Physiol. Pathophysiol. Pharmacol..

[65] Schoppet M., Al-Fakhri N., Franke F.E., Katz N., Barth P.J., Maisch B., Preissner K.T., Hofbauer L.C. (2004). Localization of osteoprotegerin, tumor necrosis factor-related apoptosis-inducing ligand, and receptor activator of nuclear factor-kappaB ligand in Mönckeberg’s sclerosis and atherosclerosis. J. Clin. Endocrinol. Metab..

[66] Cottin Y., Issa R., Benalia M., Mouhat B., Meloux A., Tribouillard L., Bichat F., Rochette L., Vergely C., Zeller M. (2021). Association between Serum Osteoprotegerin Levels and Severity of Coronary Artery Disease in Patients with Acute Myocardial Infarction. J. Clin. Med..

[67] Ma T., Zhao J., Yan Y., Liu J., Zang J., Zhang Y., Ruan K., Xu H., He W. (2023). Plasma osteoprotegerin predicts adverse cardiovascular events in stable coronary artery disease: The PEACE trial. Front. Cardiovasc. Med..

[68] Forde H., Davenport C., Harper E., Cummins P., Smith D. (2018). The role of OPG/RANKL in the pathogenesis of diabetic cardiovascular disease. Cardiovasc. Endocrinol. Metab..

[69] Marques G.L., Hayashi S., Bjällmark A., Larsson M., Riella M., Olandoski M., Lindholm B., Nascimento M.M. (2021). Osteoprotegerin is a marker of cardiovascular mortality in patients with chronic kidney disease stages 3-5. Sci. Rep..

[70] Guañabens N., Enjuanes A., Alvarez L., Peris P., Caballería L., Jesús Martínez de Osaba M., Cerdá D., Monegal A., Pons F., Parés A. (2009). High osteoprotegerin serum levels in primary biliary cirrhosis are associated with disease severity but not with the mRNA gene expression in liver tissue. J. Bone Miner. Metab..

[71] Fahrleitner-Pammer A., Dobnig H., Piswanger-Soelkner C., Bonelli C., Dimai H.P., Leb G., Obermayer-Pietsch B. (2003). Osteoprotegerin serum levels in women: Correlation with age, bone mass, bone turnover and fracture status. Wien. Klin. Wochenschr..

[72] Ostrowska Z., Ziora K., Oświęcimska J., Marek B., Świętochowska E., Kajdaniuk D., Strzelczyk J., Cieślicka A., Wołkowska-Pokrywa K., Kos-Kudła B. (2015). Selected pro-inflammatory cytokines, bone metabolism, osteoprotegerin, and receptor activator of nuclear factor-kB ligand in girls with anorexia nervosa. Endokrynol. Pol..

[73] van Tuyl L.H., Voskuyl A.E., Boers M., Geusens P., Landewé R.B., Dijkmans B.A., Lems W.F. (2010). Baseline RANKL:OPG ratio and markers of bone and cartilage degradation predict annual radiological progression over 11 years in rheumatoid arthritis. Ann. Rheum. Dis..

[74] Kichev A., Rousset C.I., Baburamani A.A., Levison S.W., Wood T.L., Gressens P., Thornton C., Hagberg H. (2014). Tumor necrosis factor-related apoptosis-inducing ligand (TRAIL) signaling and cell death in the immature central nervous system after hypoxia-ischemia and inflammation. J. Biol. Chem..

[75] Vitovski S., Phillips J.S., Sayers J., Croucher P.I. (2007). Investigating the interaction between osteoprotegerin and receptor activator of NF-kappaB or tumor necrosis factor-related apoptosis-inducing ligand: Evidence for a pivotal role for osteoprotegerin in regulating two distinct pathways. J. Biol. Chem..

[76] Zauli G., Rimondi E., Nicolin V., Melloni E., Celeghini C., Secchiero P. (2004). TNF-related apoptosis-inducing ligand (TRAIL) blocks osteoclastic differentiation induced by RANKL plus M-CSF. Blood.

[77] Haden S.T., Brown E.M., Hurwitz S., Scott J., El-Hajj Fuleihan G. (2000). The effects of age and gender on parathyroid hormone dynamics. Clin. Endocrinol..

[78] Steingrimsdottir L., Gunnarsson O., Indridason O.S., Franzson L., Sigurdsson G. (2005). Relationship between serum parathyroid hormone levels, vitamin D sufficiency, and calcium intake. JAMA.

[79] Kim S.M., Zhao D., Schneider A.L.C., Korada S.K., Lutsey P.L., Guallar E., Alonso A., Windham B.G., Gottesman R.F., Michos E.D. (2017). Association of parathyroid hormone with 20-year cognitive decline: The ARIC study. Neurology.

[80] Guerreiro R., Wojtas A., Bras J., Carrasquillo M., Rogaeva E., Majounie E., Cruchaga C., Sassi C., Kauwe J.S., Younkin S. (2013). TREM2 variants in Alzheimer’s disease. N. Engl. J. Med..

[81] Jonsson T., Stefansson H., Steinberg S., Jonsdottir I., Jonsson P.V., Snaedal J., Bjornsson S., Huttenlocher J., Levey A.I., Lah J.J. (2013). Variant of TREM2 associated with the risk of Alzheimer’s disease. N. Engl. J. Med..

[82] Jin S.C., Carrasquillo M.M., Benitez B.A., Skorupa T., Carrell D., Patel D., Lincoln S., Krishnan S., Kachadoorian M., Reitz C. (2015). TREM2 is associated with increased risk for Alzheimer’s disease in African Americans. Mol. Neurodegener..

[83] Wang H.K., Hung C.M., Lin S.H., Tai Y.-C., Lu K., Liliang P.-C., Lin C.-W., Lee Y.-C., Fang P.-H., Chang L.-C. (2014). Increased risk of hip fractures in patients with dementia: A nationwide population-based study. BMC Neurol..

[84] Yu J.T., Jiang T., Wang Y.L., Wang H.-F., Zhang W., Hu N., Tan L., Sun L., Tan M.-S., Zhu X.-C. (2014). Triggering receptor expressed on myeloid cells 2 variant is rare in late-onset Alzheimer’s disease in Han Chinese individuals. Neurobiol. Aging.

[85] Sell G.L., Schaffer T.B., Margolis S.S. (2017). Reducing expression of synapse-restricting protein Ephexin5 ameliorates Alzheimer’s-like impairment in mice. J. Clin. Investig..

[86] Ding X., Chen Y., Guo C., Fu Y., Qin C., Zhu Q., Wang J., Zhang R., Tian H., Feng R. (2023). Mutations in ARHGEF15 cause autosomal dominant hereditary cerebral small vessel disease and osteoporotic fracture. Acta Neuropathol..

