# Supplementary material for: Potential Biological and Genetic Links Between Dementia and Osteoporosis: A Scoping Review
Source: Geriatrics (Basel). 2025 Jul 20;10(4):96. doi: 10.3390/geriatrics10040096 (PMC12333915; doi:10.3390/geriatrics10040096)
Supplement: Supplementary file 1 [file geriatrics-10-00096-s001.zip › geriatrics-3701398-supplementary.pdf]

Supplementary material

Potential Biological and Genetic Links Between Dementia and Osteoporosis: A scoping review

Abayomi N. Ogunwale[1], Paul E. Schulz[1], Jude K. des Bordes[1], Florent Elefteriou[2], Nahid J. Rianon[1*]

1 UTHealth McGovern Medical School, Houston, TX, 77030 USA

2 Baylor College of Medicine, Houston TX, 77030 USA

* Correspondence: Nahid.J.Rianon@uth.tmc.edu

Table S1 Search Strategy Employed in Primary Database (Ovid Medline)

**1**	aged/ or exp "aged, 80 and over"/ or middle aged/
**2**	("middle age" or "middle-age" or midlife or "mid-life" or age* or elderly or retire* or octogenarian* or nonagenarian* or centenarian* or "baby boomer*" or "senior citizen*" or ((nursing or retirement) adj3 "home resident*") or "over 50").ti,ab,kw.
**3**	1 or 2
**4**	exp Osteoporosis/
**5**	(Osteoporo* or "Bone Loss*" or "Bone-Loss" or Boneloss or "Age-Related Bone Loss*" or "Perimenopausal Bone Loss*" or "Peri-menopausal Bone Loss*" or "Postmenopausal Bone Loss*" or "Post-menopausal Bone Loss*" or (("age-related" or Perimenopausal or "Peri-menopausal" or Postmenopausal or "Post-menopausal") adj3 ("Bone Loss*" or "Bone-loss*" or Boneloss* or Osteoporo*))).ti,ab,kw.
**6**	4 or 5
**7**	exp Dementia/
**8**	(Dementia* or Amentia* or Alzheimer* or Alzhemier* or ADRD or ((HIV or "Human Immunodeficiency Virus" or "Alzheimer-Type" or "Alzhemier-Type" or Vascular or Arteriosclerotic) adj3 Dementia*) or ((HIV or "Human Immunodeficiency Virus" or AIDS or "Acquired-Immune Deficiency Syndrome") adj3 Encephalopath*) or (("Acquired-Immune Deficiency Syndrome" or AIDS or "AIDS-Related") adj3 "Dementia Complex") or (("Human Immunodeficiency Virus" or "HIV-Associated" or "HIV-1-Associated" or "HIV-1") adj3 "Cognitive Motor Complex") or "Primary Progressive Aphasia*" or "Mesulam* Syndrome*" or (("Creutzfeldt-Jakob" or "Jakob-Creutzfeldt") adj3 (Syndrome* or Disease*)) or "subacute Spongiform Encephalopath*" or "V-CJD" or VCJD or "Binswanger* Disease*" or "Binswanger Encephalopath*" or "Subcortical Leukoencephalopath*" or "Subcortical Arteriosclerotic Encephalopath*" or "Kosaka-Shibayama Disease" or "Frontotemporal Lobar Degeneration*" or FTLD or FTLDs or (Huntington* adj3 (Chorea or Disease)) or "Chronic Progressive Hereditary Chorea" or "Kluver Bucy Syndrome" or "Temporal Lobectomy Behavior Syndrome" or "Diffuse Lewy Body Disease*" or "Cortical Lewy Body Disease*").ti,ab,kw.

Table S2 Summary of Clinical Studies Included in the Review

**Author, year**	**Biomarker or gene**	**Database and Patient Characteristics**	**Study Aim**	**Main finding**
Booth[11] 1999	*APOE4*	335 men and 553 women members of the Framingham Heart Study cohort in 1988-89 Population average 75.2 years (Men 75.1 ± 4.93 and Women 75.3 ± 4.83) years	Evaluate the relationship between dietary vitamin K, *APOE* genotype, BMD and fracture risk in older adults in the Framingham Heart Study.	Relative risk of fracture risk significantly lower in individuals reporting highest quartile of vit k intake. However, neither vit K1 intake nor APOE4 status were associated with BMD and hip fracture.
Cauley [12] 1999	*APOE4*	1750 Women enrolled at the Pittsburgh Clinical Center for Study of Osteoporotic Fractures	Examine the longitudinal relationship between presence of *APOE4* Allele	Risk of hip and wrist fractures is higher in women with at least one APOE4 allele following

**Author, year**	**Biomarker or gene**	**Database and Patient Characteristics**	**Study Aim**	**Main finding**
		Mean age 71.3 years<br>*APOE* genotype distribution: *E*2/E2 (0.5%, n = 9), *E*3/E3 (69.7%, *n* = 1220), E3/E4 (12.5% *n* = 219), E3/E2 14.3%, *n* = 250; E4/E2 (1.3%, *n* = 23), E4/E4 (1.7%, *n* = 29)	and BMD in postmenopausal women.	adjustments for confounders like age, falls, BMD, cognitive impairments.
Dick [13] 2002	*APOE4*	1332 healthy elderly randomly recruited from the community<br>*APOE4* +, n = 1024, Age (75.2±2.7) years<br>*APOE4* negative, n = 308, Age (75± 2.7) years	To evaluate the association between presence of *APOE4* allele and both bone quantitative ultrasound measures and fractures (incident and prevalent).	Presence of the APOE4 allele is associated with 2% lower hip BMD and lower quantitative ultrasound measures at the calcaneus, but not

**Author, year**	**Biomarker or gene**	**Database and Patient Characteristics**	**Study Aim**	**Main finding**
				associated with prevalent or incident non-traumatic fractures.
Efstathiadou[14] 2004	*APOE4*	147 healthy peri- and postmenopausal women Mean age: 54.3 ± 7.8 years *APOE* genotype distribution: E2/2 0.7% (n = 1), E2/3 5.4% (n = 8), E2/4 2% (n = 3), E3/3 73.5% (n = 108), E3/4 16.3% (n = 24), E4/4 2% (n = 3).	Assess the impact of *APOE* polymorphism, and specifically presence of *APOE4* allele on metabolic bone markers and spinal BMD in a cohort of Greek women.	Presence of APOE4 allele was not associated with any change in spinal BMD and markers of bone metabolism (osteocalcin, urinary pyridinoline/creatinine and deoxypyridinoline/creatinine ratios) in the cohort

Author, year	Biomarker or gene	Database and Patient Characteristics	Study Aim	Main finding
				of peri-and post-menopausal Greek women
Heikkinen [15] 2000	*APOE4*	352 early postmenopausal women recruited from the Kuopio Osteoporosis Risk Factor and Prevention Study (n= 13,000) *APOE4* negative women, n= 234, Average age: (52.7 ± 0.1), *APOE4* Positive women, n= 118, Average age: (52.7 ± 0.2) years	To investigate the relationship between *APOE* genotype and postmenopausal bone loss and to evaluate the effect of APOE genotype on the effect of hormone replacement therapy (HRT) on postmenopausal bone.	APOE genotype was not associated with the lumbar or femoral neck BMD. Femoral neck BMD remained stable and lumbar spine BMD increased in individuals with HRT compared to those without, but APOE genotype had no effect

Author, year	Biomarker or gene	Database and Patient Characteristics	Study Aim	Main finding
				on HRT driven BMD changes.
Johnston [16] 1999	*APOE4*	899 community-dwelling older adults in southwestern Pennsylvania selected from the Monongahela Valley Independent Elders Survey (MoVIES) prospective community study.<br>Average age 76.2 ± 4.9 years	To examine the association between *APOE4* allele and risk of hip fracture in older community-based patients.	Participants having at least one APOE4 allele had twice the risk of hip fracture (OR 2.1, CI: 0.9-4.7) following adjustments for dementia and fall history.

**Author, year**	**Biomarker or gene**	**Database and Patient Characteristics**	**Study Aim**	**Main finding**
		Allele frequencies *APOE2* (0.068); *APOE3* (0.819), and *APOE4* (0.112).		
Kohlmeier [17] 1998	*APOE4*	123 women and 96 men recruited from 6 of the 12 shifts at three hemodialysis centers for adults in Berlin, Germany. Mean age 65.2 years *APOE* genotype distribution: E2/2: 3.2%, E2/3: 14.2% E2/4: 0.5%, E3/3: 63.5%, E3/4: 17.8%, E4/4: 0.9%	To determine the association between *APOE* genotypes and bone fracture risk in hemodialysis patients.	Participants with E3/4 and E4/4 reported a higher number of bone fractures (17.1 %) compared to those with the E2/3 and E2/2 genotypes (5.3%), but the difference was not statistically significant ($P < 0.1$).

**Author, year**	**Biomarker or gene**	**Database and Patient Characteristics**	**Study Aim**	**Main finding**
Pluijm [9] 2002	*APOE4*	604 participants aged 55-85 years from west and south Netherland selected from the Longitudinal Aging Study Amsterdam (LASA).<br>*APOE* genotype distribution<br>E2/E2 (1.2%), E2/E3 11.8%<br>E3/E3 61.9%, E2/E4 2.6%<br>E3/E4 19.9, E4/e4 2.6	To determine the association between of *APOE E4* and BMD, quantitative ultrasound (QUS) measurements, bone turnover and fracture risk in older men and women.	Among women participants, there was an association between presence of APOE4 and lower femoral neck BMD<br>ApoE epsilon4 was associated with significantly lower BMD at the femoral neck and lower trochanter, and lower total body BMC .<br>Presence of APOE4 was also associated with a

Author, year	Biomarker or gene	Database and Patient Characteristics	Study Aim	Main finding
				higher risk of severe vertebral deformities Among men, the association between APOE Status and both hip BMD and QUS was mediated by age. Presence of APOE4 in younger men (65-69 years) was associated with lower BMD values.

**Author, year**	**Biomarker or gene**	**Database and Patient Characteristics**	**Study Aim**	**Main finding**
Schoofs [18] 2009	*APOE*	5857 subjects (2560 men; 3297 women) recruited in the prospective population-based Rotterdam Study *APOE* genotype distribution (%): E2/2 (0.8%), E3/E2 (13%), E3/E3 (57.9%), E4/E2 (2.6%), E4/E3 (23.3%) and E4/E4 (2.4%) Ages of participants stratified by E4 carrier status:	To assess the association between *APOE* genotype and BMD, bone loss, and incident fractures.	There was no significant difference in adjusted risks for age- and BMI-related fracture in participants with and without APOE4.

Author, year	Biomarker or gene	Database and Patient Characteristics	Study Aim	Main finding
		Non E4 allele carriers: men (68.4 ± 8.3) years, women (68.1 ± 7.9) years<br>E4 allele carriers: men (70.6.4 ± 9.5) years, women (69.9 ± 9.4) years		
Shiraki [19] 1997	*APOE4*	284 Postmenopausal Japanese women (n= 284)<br>Ages 47–82 years (64.0 ± 1.0) years<br>*APOE* genotype distribution: E2/3 (9.9%, *n* = 28), E3/3 (66.5%, *n* = 189), E2/4 (1.8%, *n* = 5),	Evaluate associations between *APOE* phenotype and BMD in postmenopausal Japanese women.	A gene-dose, independent association exists between presence of the APOE4 allele and both low lumbar spine and whole-body BMD and Z scores.

**Author, year**	**Biomarker or gene**	**Database and Patient Characteristics**	**Study Aim**	**Main finding**
		E3/4 (19.7%, *n* = 56), and E4/4 (2.1%, *n* = 6).		
Souza [20] 2017	*APOE4*	529 randomly recruited Brazilian women aged over 50 years and post-menopausal for at least 12 months.<br>Average age 64.1 ± 8.0 years<br>*APOE* genotype distribution<br>E2/2: n=17 (7.9%), E2/3: n= 54 (25.2%), E2/4: n = 13 (6%), E3/3: n= 68(31.8%),	To determine relationship between *APOE* genotype and clinical osteoporosis.	There was an association between APOE3 allele and both higher BMDs and higher serum concentrations of osteocalcin and alkaline phosphatase.<br>There was also an association between APOE2 and APOE4 alleles and lower BMD and as higher levels of

Author, year	Biomarker or gene	Database and Patient Characteristics	Study Aim	Main finding
		E3/4: n= 58 (27.1%) E4/4: n= 4 (0.2%)		serum C-terminus collagen peptide and urinary deoxipyridinolines, biomarkers for bone resorption. These associations with lower BMD and bone resorption biomarkers APOE2 or APOE4 alleles disappeared in the presence of the APOE3 allele.

Author, year	Biomarker or gene	Database and Patient Characteristics	Study Aim	Main finding
Stefanidou [29] 2021	*APOE4*	870 male and 1035 female offsprings of the original members of the Framingham Heart Study cohort<br>Mean age: Men 66.28 ± 8.85 years, Women 65.64± 9.17 years	To investigate the relationship between BMD and neuroimaging biomarkers of brain aging and cognitive function in older adults without dementia	There is a positive association between higher femoral neck BMD and reduced brain white matter burden, as well as better, domain-specific, cognitive performance among offspring participants of the Framingham Study.
Wong [31] 2005	*APOE2, E3, E4*	457 women aged 55–79years, and 235 men aged 70–79 years recruited from	Examine the association between the presence of the *APO E4, E2*, and *E3*	No association observed between presence of

Author, year	Biomarker or gene	Database and Patient Characteristics	Study Aim	Main finding
		the Shatin district, Hong Kong<br>*APOE* genotype distribution<br>E2/2 (1%), E3/E2 (15.6%), E3/E3 (68.9%), E4/E2 (0.4%), E4/E3(13.6%) and E4/E4(0.4%)	genotypes and BMD in Chinese men and women	APOE4 allele and BMD z scores in the cohort of Chinese men and women. APOE2 is associated with BMD at femoral neck(p=0.02) and the spine (0.031) in the women aged 70-79 years.
von Mühlen [30] 2001	*APOE4*	596 men and 332 community-dwelling women aged 45–95 years enrolled in the Rancho Bernardo study	To examine the relationship between presence of the *APOE4* allele and BMD, bone loss and fracture risk	Presence of the APOE4 allele is not associated with BMD in men and women participants, nor the annual rate of BMD

Author, year	Biomarker or gene	Database and Patient Characteristics	Study Aim	Main finding
				change, bone loss and fracture risk
Braverman [21] 2009	PTH	92 patients aged 18- 90 years from PATH (an integrative care/research center). Age (mean ± SD) years = (58.85 ± 15.47) years	To evaluate the association between serum parathyroid hormone (PTH) and brain processing speed and bone density	Age-related PTH elevation is directly associated with an index of brain processing speed (P300 latency) and inversely associated with bone mineral density.
Emanuele [22] 2004		39 patients with VaD, 36 patients with AD, and 39 individuals without dementia matched by age and gender.	To compare plasma levels of OPG in patients with VaD and Alzheimer's disease (AD), and age- and gender-matched controls	There was a positive association between plasma OPG age in individuals with

Author, year	Biomarker or gene	Database and Patient Characteristics	Study Aim	Main finding
		VaD patients: 24 females and 15 males. mean age + SD = 77.9+7.3 years (range 64- 98 years)<br><br>AD patients: 22 females and 14 males. mean age + SD = 77.7+6.6 years (range 63-93 years)	without dementia, and to examine differences in plasma OPG levels between individuals with VaD and AD.	and without dementia. An independent association between plasma OPG and VaD persisted even after adjusting for age, gender and *APOE4*allele.
Luckhaus [24] 2009	OPG	Individuals with mild cognitive impairment (n=19), mild Alzheimer's disease (n=20), and age-	Identify molecular pathology spanning osteoporosis and dementia	Osteocalcin is high in controls with osteoporosis but higher

Author, year	Biomarker or gene	Database and Patient Characteristics	Study Aim	Main finding
		matched cognitively normal controls (n=8) recruited from outpatient psychiatry clinic in Duesseldorf, Germany<br>Ages of participants were: Cognitively normal controls (72.4 ± 6.1) years<br>Mild cognitive impairment (67.26±10.9) years<br>AD (70.35±8.23) years		in patients with mild AD but not MCI. OPG level is not significantly different between the groups. There are increased levels of markers of bone breakdown and remodeling that is not a result of vitamin D level in the patients with mild AD,

**Author, year**	**Biomarker or gene**	**Database and Patient Characteristics**	**Study Aim**	**Main finding**
				compared the patients with MCI.
Ross [27] 2018	Dkk1, TRAIL	88 female and 15 male community-dwelling older adults with memory complaints but no formal diagnosis of cognitive impairment.<br>Mean age of participants: 80.3 years (SD = 7.7)	To evaluate the association between serum bone biomarkers and cognition	There were associations between serum levels of TRAIL (p<0.001), DKK1 (p=0.14), CTX-1 (p=0.46) and rate of change of markers of cognition over 18 months.
Ma [25] 2020	*TREM2*	279 LOAD patients recruited from three university affiliated hospitals - Ruijin Hospital,	To evaluate the association between *TREM2* polymorphism and late	No *TREM2* variant was detected in the population of cases, and

Author, year	Biomarker or gene	Database and Patient Characteristics	Study Aim	Main finding
		Nanjing Brain Hospital and Shanghai Pu Tuo People's Hospital) and 346 age-, sex-, and ethnical origin- matched controls with no neurologic impairment enrolled from the local community from 2008 to 2012.<br>Mean age: LOAD patients 72.42 ± 8.53 years, Controls 72.75 ± 8.16 years<br>Participants with no *APOE4* allele (*APOE4 -/-):* LOAD patients 156	onset AD in Chinese Han population	presence of *APOE4* patients was associated with late onset dementia.

**Author, year**	**Biomarker or gene**	**Database and Patient Characteristics**	**Study Aim**	**Main finding**
		(55.9%), Controls 288 (83.2%)<br>Participants with one *APOE4* allele (*APOE4* -/+): LOAD patients 96 (34.4%), Controls 53 (15.3%)<br>Participants with double *APOE4* alleles (*APOE4* +/+): LOAD patients 27 (9.7%), Controls 5 (1.6%)<br>Participants with TREM2 (rs75932628-T) mutation: LOAD patients 0, Controls 0		

**Author, year**	**Biomarker or gene**	**Database and Patient Characteristics**	**Study Aim**	**Main finding**
Stapledon [28] 2021	*APP*, *APLP2*, *BACE1*, *NGF*	66 patients getting hip arthroplasty for neck of femur fractures at the Royal Adelaide Hospital (RAH) Adelaide, South Australia, between July 2018 and July 2019. Participants' ages ranged from 58 to 96 years. Mean age was 81.9 ± 9.15 years	To assess for the expression of neurotropic or dementia-related genes in patients having total hip replacement surgery on account of neck of femur fracture (NOF).	Serum levels of dementia-related genes - APP, *APLP2, BACE1, NGF- a*re significantly correlated with levels of RANKL, *TRAP*, as well as the RANKL: OPG mRNA ratio at the time of NOF.
Pan [26] 2022	CSF t-tau and p-tau$_{181}$	1628 participants selected from the Alzheimer's Disease Neuroimaging Initiative (ADNI) database	Evaluate the association between osteoporosis and AD related CSF markers.	There is association between osteoporosis and CSF total tau and p-tau181 and but not

**Author, year**	**Biomarker or gene**	**Database and Patient Characteristics**	**Study Aim**	**Main finding**
				with CSF amyloid beta level or brain entorhinal or hippocampal volumes.
Zhang [32] 2022	Aβ	Cohort 1: 62 individuals with no osteopenia and 103 individuals with osteopenia recruited from Xiangyang Central Hospital.<br><br>Cohort 2: Thirty-three cognitively normal individuals and 39	To compare markers of cognition in older adults with and without osteopenia.<br><br>Assess correlation between BMD, MSSE and CSF Aβ 42/40 in older adults with and without AD.	BMD correlates with cognitive indexes like MMSE ($p<0.001$), AVLT DR ($p=0.002$) and plasma Aβ 40/42 ($p=0.043$) in individuals with osteopenia.<br>BMD, AVLT-DR, MMSE, Aβ 42, CSF Aβ 42/40 are

Author, year	Biomarker or gene	Database and Patient Characteristics	Study Aim	Main finding
		individuals with AD recruited from Xiangyang Central Hospital.		significantly lower in participants with AD compared to non-AD participants.<br>BMD correlates positively with MMSE ($p<0.001$) and CSF Aβ 42/40 ($p<0.001$) in participants with AD
Liu [23] 2023	Brain-derived, and plasma-derived	20 individuals (8 males and 12 females) with AD and 20 healthy (8 males and 12 females) age- and	Evaluate the effect of bone derived and plasma derived EVs on bone formation, bone loss and marrow fat deposition.	Administration of brain-derived and plasma-derived EVs from AD patients were associated

Author, year	Biomarker or gene	Database and Patient Characteristics	Study Aim	Main finding
	extracellular vesicles	gender- matched controls.<br>Mean age: AD Cases (57.94 ± 8) years, Controls (61.78 ± 5.73) years		with impaired osteogenesis and increased bone fat deposition in vitro.
